# Clearance and Toxicity of Recombinant Methionyl Human Glial Cell Line-Derived Neurotrophic Factor (r-metHu GDNF) Following Acute Convection-Enhanced Delivery into the Striatum

**DOI:** 10.1371/journal.pone.0056186

**Published:** 2013-03-20

**Authors:** Hannah Taylor, Neil Barua, Alison Bienemann, Marcella Wyatt, Emma Castrique, Rebecca Foster, Matthias Luz, Christian Fibiger, Erich Mohr, Steven Gill

**Affiliations:** 1 School of Clinical Sciences, University of Bristol, Bristol, United Kingdom; 2 Department of Neurosurgery, Frenchay Hospital, Bristol, United Kingdom; 3 School of Clinical Sciences, University of Bristol, Bristol, United Kingdom; 4 MedGenesis Therapeutix Inc., Victoria, British Columbia, Canada; University of Florida, United States of America

## Abstract

**Background:**

Despite promising early results, clinical trials involving the continuous delivery of recombinant methionyl human glial cell line-derived neurotrophic factor (r-metHuGDNF) into the putamen for the treatment of Parkinson's disease have shown evidence of poor distribution and toxicity due to point-source accumulation. Convection-enhanced delivery (CED) has the potential to facilitate more widespread and clinically effective drug distribution.

**Aims:**

We investigated acute CED of r-metHuGDNF into the striatum of normal rats in order to assess tissue clearance, toxicity (neuron loss, gliosis, microglial activation, and decreases in synaptophysin), synaptogenesis and neurite-outgrowth. We investigated a range of clinically relevant infused concentrations (0.1, 0.2, 0.6 and 1.0 µg/µL) and time points (2 and 4 weeks) in order to rationalise a dosing regimen suitable for clinical translation.

**Results:**

Two weeks after single dose CED, r-metHuGDNF was below the limit of detection by ELISA but detectable by immunohistochemistry when infused at low concentrations (0.1 and 0.2 µg/µL). At these concentrations, there was no associated neuronal loss (neuronal nuclei, NeuN, immunohistochemistry) or synaptic toxicity (synaptophysin ELISA). CED at an infused concentration of 0.2 µg/µL was associated with a significant increase in synaptogenesis (p<0.01). In contrast, high concentrations of r-metHuGDNF (above 0.6 µg/µL) were associated with neuronal and synaptic toxicity (p<0.01). Markers for gliosis (glial fibrillary acidic protein, GFAP) and microglia (ionized calcium-binding adapter molecule 1, Iba1) were restricted to the needle track and the presence of microglia had diminished by 4 weeks post-infusion. No change in neurite outgrowth (Growth associated protein 43, GAP43, mRNA) compared to artificial cerebral spinal fluid (aCSF) control was observed with any infused concentration.

**Conclusion:**

The results of this study suggest that acute CED of low concentrations of GDNF, with dosing intervals determined by tissue clearance, has most potential for effective clinical translation by optimising distribution and minimising the risk of toxic accumulation.

## Introduction

Parkinson's Disease (PD) is characterised by impairment of motor function largely caused by the loss of dopaminergic neurons in the substantia nigra. Since its discovery in 1993 [1], glial cell line-derived neurotrophic factor (GDNF) has shown consistent potential as a neuroprotective and neurorestorative therapy in a succession of *in vitro* [1]–[3]; [4]–[6] and *in vivo* studies in the 6-hydroxydopamine (6-OHDA) rat model of PD [4]–[6] and in aged and 1-methyl-4-phenyl-1,2,3,6-tetrahydropyridine-lesioned non-human primates (NHPs) [7]–[10].

Following the success of these pre-clinical studies, our research group undertook the first open-label study of continuous intraputaminal delivery of recombinant methionyl human glial cell line-derived neurotrophic factor (r-metHuGDNF) via stereotactically-placed microcatheters attached to a subcutaneous infusion pump [11]. This study enrolled five patients with PD symptoms poorly controlled by medical treatment. All five patients demonstrated improvement in both clinical and fluorine-18 dihydroxyphenylalanine positron emission tomography (18F-dopa PET) imaging parameters. One patient receiving unilateral infusion of r-metHuGDNF died of causes unrelated to the study, and post mortem examination confirmed that infusion of r-metHuGDNF resulted in a marked increase in tyrosine hydroxylase-positive nerve fibres, and possibly neuronal sprouting in the substantia nigra [12].

A second open-label study by Slevin *et al.* enrolled ten patients, and showed reductions in Unified Parkinson's Disease Rating Scale (UPDRS) scores as well as improvements in postural stability, dyskinesias and end-of-dose fluctuations [13]. Success in these two open-label studies led to the commencement of a randomised controlled trial. This multicentre trial randomised 34 patients to receive either bilateral infusions of r-metHuGDNF into the putamen at a dose of 15 µg/putamen/day or placebo [14]. At six months, patients receiving r-metHuGDNF had failed to demonstrate the predetermined level of clinical improvement required to achieve statistical significance despite improvements in PET imaging parameters.

Furthermore, asymptomatic neutralising antibodies were detected in four patients [15] and a further nine patients developed serious device-related adverse effects [16]. At the same time, a six month chronic intraputamenal infusion toxicity study in rhesus monkeys showed unexpected cerebellar lesions in a small number of animals that received a very high dose of r-metHuGDNF [17]. The disappointing results of the randomised Phase II study taken in conjunction with the six-month toxicity study in primates led to withdrawal of r-metHuGDNF and cessation of clinical trials.

There followed an in depth analysis of the factors which had resulted in failure of the phase II trial. Salvatore *et al.* analysed the distribution of Iodine^125^-GDNF in the putamen of rhesus monkeys when delivered using the same delivery system and protocol as in the phase II study [18]. Analysis of r-metHuGDNF distribution within the putamen by immunohistochemistry and local concentration measurements revealed significant variability, with the majority of r-metHuGDNF restricted to the immediate vicinity of the catheter tip [18]. On the basis of their data, the authors estimated that drug bioavailability was limited to a small portion (2–9%) of the human putamen in the clinical trial using this catheter and infusion protocol.

GDNF signals through a multicomponent receptor system comprising a high-affinity ligand-binding co-receptor GFRα (GDNF family receptor α-component) and the RET receptor tyrosine kinase [19] [20]. These receptors are highly expressed in the midbrain, specifically, in the dopaminergic and non-dopaminergic neurons of the substantia nigra, and to a lesser extent in the substantia nigra pars reticulata and the ventral tegmental area [21]. Heparin sulphate proteoglycans also participate in the signalling of GDNF [22]. The avid binding of r-metHuGDNF to heparin binding sites in the extracellular matrix of the brain parenchyma significantly limits its diffusivity [23]. One way to overcome limited diffusivity would be to use GDNF mutants which have lower affinity for heparin sulphate proteoglycans [24]. However, the effects of these mutants on the GDNF receptor system, and thus the neurotrophic influence of GDNF, are not fully known. Convection-enhanced delivery (CED) has emerged as a novel neurosurgical technique which has the potential to achieve more effective coverage of the putamen. CED describes a direct method of drug delivery to the brain through very fine microcatheters. By establishing a pressure gradient at the tip of the infusion catheter, CED confers several advantages over conventional drug injection techniques, in particular, homogeneous drug distribution through large and clinically-relevant brain volumes [25].

Whilst CED has the potential to improve intraputaminal drug distribution, further strategies are likely to be required to prevent accumulation of r-metHuGDNF and toxicity. The aim of the current study was to characterise the clearance and toxicity of r-metHuGDNF following acute CED into the striatum of normal aged rats in order to determine whether acute infusions might have greater translational potential than previously employed continuous infusion regimens.

## Methods

### 2.1 Convection-enhanced delivery procedures

All animal work was performed in accordance with the UK Animal Scientific Procedures Act 1986 and was covered by both Project and Personal licences that were issued by the Home Office and these were also reviewed and approved by the University of Bristol ethical committee (project licences 30/2353 & 30/2902). All efforts were made to minimise animal use and suffering. Adult male Wistar rats (Charles River, Margate, UK, 225 to 275 g) were anaesthetised with intraperitoneal ketamine (Ketaset; 60 mg/kg, Pfizer Animal Health, Sandwich, UK) and medetomidine (Dormitor; 0.4 mg/kg, Pfizer), and then placed in a stereotactic frame (Stoelting, Illinois, USA). A midline skin incision was made from glabella to occiput to expose bregma. Bilateral burr holes were drilled using a 2 mm drill. All CED procedures were performed using a custom-made catheter with an outer diameter of 0.22 mm and inner diameter of 0.15 mm, composed of fused silica with a laser cut tip. The cannula was attached to a 1 ml syringe (Hamilton, Bonaduz, Switzerland) connected to a rate-controlled microinfusion pump (World Precision Instruments Inc., Sarasota, FL, USA) and the tip placed at stereotactic co-ordinates derived from the Paxinos and Watson stereotactic rat brain atlas (0.5 mm anterior and 3 mm lateral to bregma, depth 5.0 mm), in order to target the striatum [26].

A total volume of 5 µL r-metHuGDNF (MedGenesis Therapeutix Inc., Victoria, BC, Canada) at infused concentrations of 0.1, 0.2, 0.6, and 1.0 µg/µL diluted in aCSF (MedGenesis Therapeutix Inc.) or aCSF vehicle alone, was delivered into the striatum. R-metHuGDNF is a synthetic, 135-amino acid form of GDNF produced in E. coli. Its sequence differs from native human GDNF by the addition of an N-terminal methionine residue. In solution, r-metHuGDNF forms a homodimer with a molecular weight of approximately 30.4 kDa. The biological activity of r-metHuGDNF is determined by means of a cell-line based mitogenic assay.

CED procedures were performed at an infusion rate of 1.0 µL/min. On completion of CED the cannula was left *in situ* for 10 min to minimise reflux, then withdrawn at a rate of 1 mm/min. For “needle only” controls, an empty cannula was inserted into the striatum, left in situ for 10 min, and then withdrawn at 1 mm/min. The wound was closed with 4/0 Vicryl, and a dose of intramuscular buprenorphine (Centaur Services, Castle Cary, UK) was administered (30 µg/kg). The anaesthetic was reversed with 0.1 mg/kg i.p. atipamezole hydrochloride (Pfizer) in recovery procedures. Rats were euthanised by anaesthetic overdose with an intraperitoneal injection of 1 ml pentobarbital (Euthatal; Merial Animal Health, Harlow, UK) at pre-defined time-points following CED (2 weeks or 4 weeks). For immunohistochemical analysis (IHC), animals were transcardially perfused with 4% paraformaldehyde. Brains were removed and placed in 4% paraformaldehyde for 24 h, then cryoprotected in 30% sucrose. For quantitative real time polymerase chain reaction (Q-PCR) and enzyme-linked immuno-sorbent assay (ELISA), brains were explanted and rapidly frozen at −80°C until required. See table 1 for hemisphere numbers for each infused concentration of r-metHuGDNF at each time point.

**Table 1:** Number of hemispheres infused with r-metHuGDNF for qPCR, ELISA, and immunohistochemical analysis.

Infusate	Technique	Number of hemispheres infused, 2 weeks	Number of hemispheres infused, 4 weeks	Number of animals used
ACSF	QPCR	3	5	
	ELISA	3	5	
	IHC	2	4	11
0.1 µg/µL	QPCR	4	5	
	ELISA	4	5	
	IHC	2	2	11
0.2 µg/µL	QPCR	3	4	
	ELISA	3	4	
	IHC	2	2	9
0.6 µg/µL	QPCR	3	5	
	ELISA	3	5	
	IHC	2	2	10
1.0 µg/µL	QPCR	6	4	
	ELISA	6	4	
	IHC	2	2	12
Needle only	QPCR	3	2	
	ELISA	3	2	
	IHC	2	2	7

### 2.2 GDNF Sandwich ELISA

Striatal tissue punches of approximately 100 mg were dissected from hemispheres infused with varying concentrations of r-metHuGDNF in aCSF or aCSF alone. Tissue was placed into lysis buffer containing 137 mM Sodium chloride (Melford, Suffolk, UK), 20 mM Tris (pH 8.0; Melford, Suffolk, UK), 1% Triton-x-100 (Sigma Aldrich, Dorset, UK), 10% glycerol (Melford, Suffolk, UK), 1 mM phenylmethanesulfonylfluoride (PMSF) (Sigma Aldrich), 0.5 mM sodium vanadate (Sigma Aldrich), 10 µg/mL aprotinin (proteolytic enzyme inhibitor; Sigma Aldrich), 1 µg/mL leupeptin (protease inhibitor; Sigma Aldrich) and homogenised. Samples were centrifuged at 10,000× g for 10 minutes, and the supernatant collected. Samples were acidified with 1N hydrochloric acid VWR, Lutterworth, UK to facilitate quantification of total human GDNF. Following this, tissue r-metHuGDNF levels were measured using a commercially available GDNF E_max_® Immunoassay system (Promega, Madison, WI, USA). In addition, a standard curve using r-metHuGDNF was completed and values equivalent to the GDNF E_max_® Immunoassay system standard were obtained. Absorbance was measured using a plate reader (Multiskan Ascent, Thermo Scientific, Loughborough, UK) at 450 nm. Results were expressed as pg of total human GDNF per mg of tissue or total protein (Pierce BCA protein assay kit from Thermo Scientific (Loughborough, UK).

### 2.3 Histology

Rat brains were cut into 35 µm thick coronal sections using a Leica CM1850 cryostat (Leica Microsystems, Wetzlar, Germany) at −20°C. For fluorescent immunohistochemistry, fixed sections were mounted on gelatine-subbed slides. Once dry, the sections were washed with phosphate buffered saline (PBS) for 5 min ×3. Sections were blocked in PBS plus 0.1% triton-x-100 containing 10% normal donkey serum (Sigma Aldrich) for 1 hour at room temperature (RT). They were then washed with 0.1% triton-x-100 in PBS for 5 min. Following washing, sections were incubated in goat anti-GDNF primary antibody (1∶250; R&D Systems, Abingdon, UK) in order to determine the presence and distribution of infused r-metHuGDNF.

Neurons, astrocytes, microglia, and apoptotic cells were identified by staining with the following primary antibodies: mouse anti-neuronal nuclei (NeuN) (1∶300; Millipore, MA, USA), rabbit anti-glial fibrillary acidic protein (GFAP) (1∶200; Merck Millipore), mouse anti-ionized calcium-binding adapter molecule 1 (Iba1) (1∶200; Abcam, Cambridge, UK) and rabbit anti-activated caspase 3 (1∶100; Millipore, MA, USA) at 4°C overnight in order to identify neuronal disruption, gliosis and activated microglia. The next day, primary antibody was removed and sections were washed with 0.1% triton-x-100 in PBS for 5 min ×3. Sections were incubated in donkey anti-goat Alexa Fluor® 488 (1∶500, Life Technologies, Paisley, UK), either alone, or with donkey anti-mouse Cy3 or donkey anti-rabbit Cy3 (1∶300; Jackson Laboratories, Sacramento, CA, USA) and 4',6-diamidino-2-phenylindole (DAPI) (1∶200 of 1 mg/mL; Sigma Aldrich) at RT for 2 hours in the dark and then washed with PBS for 5 min ×3. Sections were mounted in Fluorsave™ Reagent (Calbiochem®, Merck Millipore, Billerica, MA, USA) before viewing. Images were captured using the Stereo Investigator platform (MicroBrightField Bioscience, Williston, VT, USA) with a Leica DM5500 microscope (Leica Microsystems, Germany) and a digital camera (Microbrightfield Bioscience, Williston, VT, USA).

### 2.4 GAP43 mRNA quantitative RT-PCR

Growth associated protein 43 (GAP43) mRNA rather than tyrosine hydroxylase concentration was analysed as a marker of neurite outgrowth as changes in tyrosine hydroxylase are not always indicative of neurite outgrowth and could occur as a consequence of upregulated enzyme. Brains were removed, rapidly frozen with dry ice and stored at −80°C until required. Striatal tissue punches between 80–120 mg in wet weight were placed in 1 mL Tri Reagent solution (Applied Biosystems, Carlsbad, CA, USA California) and homogenised. Total RNA was isolated from the tissue punches according to the manufacturer's protocol (Applied Biosystems). A total of 1 µg ribonucleic acid (RNA) was converted into complementary deoxyribonucleic acid (cDNA) using the High Capacity RNA-to-cDNA kit (Applied Bio systems). A 1∶100 dilution of cDNA was used for PCR using SYBR® Green Jumpstart Taq ReadyMix (Sigma Aldrich) in a Real-Time PCR instrument (StepOne Plus PCR System, Applied Biosystems). Primer sequences were GAP43 forward, 5′-CAGGAAAGATCCCAAGTCCA-3′, reverse 5′-GAGGAAAGTGGACTCCCACA-3′; and beta-actin (endogenous control) forward, 5′GACAGGATGCAGAAGGAGATTA-3′, reverse 5′- TGATCCACATCTGCTGGAAGGT-3′ (Invitrogen Life Technologies). Dissociation curve analysis confirmed the amplification of primer specific products. RNA expression levels were determined by threshold cycle (Ct) analysis settings of StepOne software (Applied Biosystems) using the ΔCt comparative method of relative quantification.

### 2.5 Synaptophysin Sandwich ELISA

Synaptophysin ELISA was performed on rat brain homogenates at 2 and 4 weeks following CED of either r-metHuGDNF in aCSF or aCSF alone. The ELISA protocol for measuring synaptophysin concentration was based on that previously described by Siew *et al.* [27] and adapted for rat brain homogenates. Striatal tissue punches of approximately 100 mg were dissected from hemispheres infused with varying concentrations of r-metHuGDNF in aCSF or aCSF alone. Tissue samples were homogenised in lysis buffer and total protein measured using Pierce BCA protein assay kit (Thermo Scientific) as described above. 96-well plates (Nunc Maxisorp, Nunc, Rochester, NY, USA) were coated with primary rabbit anti-synaptophysin polyclonal antibody (Abcam) at a concentration of 1 µg/ml, and incubated overnight at 4°C. After 5 washes with wash buffer, non-specific binding was blocked with the addition of 1% bovine serum albumin (BSA) (Sigma Aldrich)/PBS for 2 hours. After another 5 washes, serial dilutions of recombinant synaptophysin (Abnova, Taipei City, Taiwan) or the supernatant of crude homogenates were added and incubated at room temperature for 2 hours. After 5 further washes, secondary antibody (mouse monoclonal anti-synaptophysin, Santa Cruz, CA, USA) was used at 1∶1000 in 1% BSA/PBS, and incubated for 2 hours. Tertiary antibody horse radish peroxidase-labelled anti-mouse antibody, Sigma Aldrich) was added to wells at 1∶200 after 5 washes, and incubated for 30 minutes in the dark. Peroxidase substrate (R&D Systems, Minneapolis, MN, USA) was added to wells for 5 min, and then the reaction stopped with STOP solution (R&D Systems). Plates were immediately read in a microplate reader at 450 nm. Samples were tested in triplicate on two separate occasions.

### 2.6 Statistical Analysis

Results were analysed using one-way analysis of variance (ANOVA) with Dunnett post hoc test using GraphPad Prism 4 software. Results are expressed as mean ± standard deviation (SD) and considered significant at *p*<0.05.

## Results

### 3.1 Striatal r-metHuGDNF concentration at 2 and 4 weeks post-infusion

The concentration of human GDNF in rat striatum at each of four infused concentrations of r-metHuGDNF (0.1, 0.2, 0.6, or 1.0 µg/µL) was determined by ELISA at 2 and 4 weeks post-infusion.

**Figure 1:**
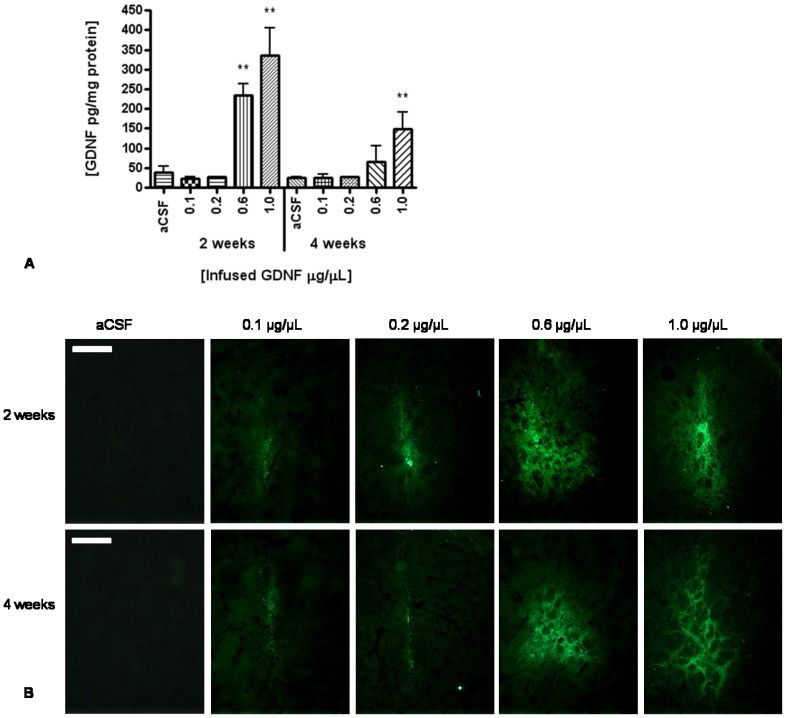
Pharmokinetics of r-metHuGDNF. (A) ELISA to show amount of total human GDNF protein (pg/mg of total protein) within the striatum after 2 weeks and 4 weeks following acute CED of 0.1, 0.2, 0.6, and 1.0 µg/µL r-metHuGDNF into the striatum. The flow rate employed was 1 µL/minute. Each bar indicates the mean ± SD. **p<0.01, r-metHuGDNF infused hemispheres versus aCSF control infused hemispheres by one-way ANOVA and Dunnett post-hoc test. (B) Representative images of immunostaining for GDNF after 2 weeks and 4 weeks following acute CED of 0.1, 0.2, 0.6, and 1.0 µg/µL r-metHuGDNF into the striatum. Scale bar = 250 µm.

CED of r-metHuGDNF at concentrations of 0.6 and 1.0 µg/µL resulted in statistically significant elevation of tissue concentrations compared to control at 2 weeks post-infusion (*p*<0.01 one-way ANOVA and Dunnett post-hoc test, Figure 1A). The highest concentration of r-metHuGDNF, 335.3±69.1 pg/mg (mean ± SD) of protein was detected at 2 weeks following infusion of 1.0 µg/µL. At 4 weeks post infusion, the tissue concentration had reduced to approximately half, 148.3±42.9 pg/mg of protein. Infusion of r-metHuGDNF at concentrations of 0.1 and 0.2 µg/µL resulted in GDNF levels below the detection limit of the ELISA at both 2 and 4 weeks, indicating either substantial clearance or breakdown of exogenous r-metHuGDNF within the initial 2 week post-infusion period.

Fluorescence immunolabeling at 2 and 4 weeks detected dose-dependent presence and distribution of r-metHuGDNF in striatum (Figure 1B). Consistent with the ELISA results, the strongest fluorescent signals were seen at infused concentrations of 0.6 and 1.0 µg/µL, although there was no appreciable difference between 2 and 4 weeks post infusion.

### 3.2 Analysis of toxicity

#### 3.2.1 Effect of r-metHuGDNF on inflammation

**Figure 2:**
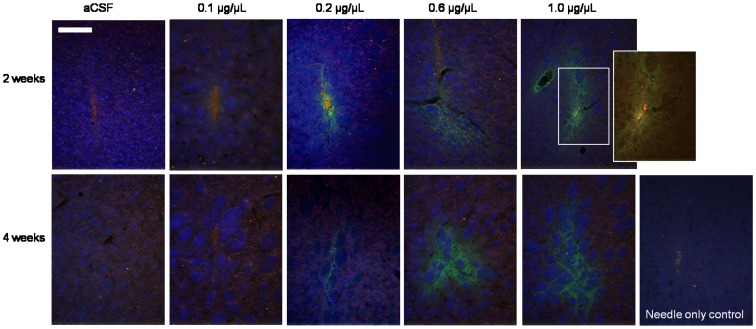
Immunostaining for Iba1, a protein upregulated during the activation of microglia. Immunostaining is shown 2 weeks and 4 weeks after r-metHuGDNF infusion. Iba1 (red) was seen in all infused concentrations at 2 weeks. The inlay shows the presence of perivascular microglia. GDNF is shown in green and nuclei marker, DAPI, in blue. Scale bar = 250 µm.

At 2 weeks post-infusion, Iba1-immunopositive cells were detectable in and around the needle track in all control (aCSF and needle only) and r-metHuGDNF infused hemispheres (Figure 2). At 4 weeks, Iba1-positive cells were no longer detectable despite the persistence of significant levels of r-metHuGDNF (as determined by IHC and ELISA), suggesting that the microglial response was mediated by mechanical damage rather than infusion of r-metHuGDNF.

#### 3.2.2 Gliotic response to r-metHuGDNF infusion

**Figure 3:**
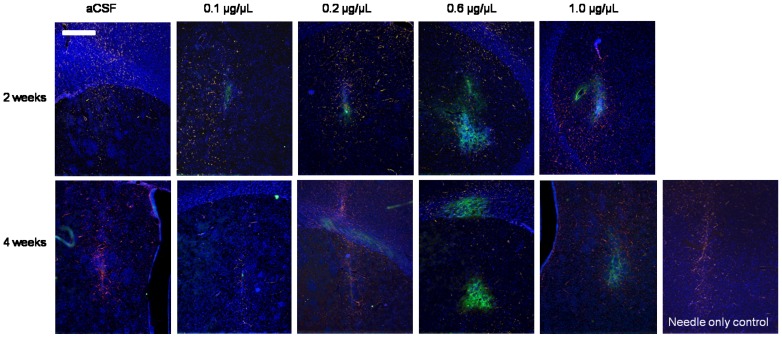
Immunostaining for Glial Fibrillary Acidic Protein (GFAP). GFAP-immunopositive cells were observed in the tissue surrounding the needle track area at both 2 and 4 weeks (Figure 3). There was no detectable correlation between the degree of gliosis adjacent to the needle track and the infused concentration of r-metHuGDNF at any time point. Scale bar = 500 µm.

GFAP-immunopositive cells were observed in the tissue surrounding the needle track area at both 2 and 4 weeks (Figure 3). There was no detectable correlation between the degree of gliosis adjacent to the needle track and the infused concentration of r-metHuGDNF at any time point, and comparable levels of gliosis were visible in all control hemispheres (aCSF and needle only; Figure 3). These data suggest that the gliotic response occurred in response to cannula insertion.

#### 3.2.3 Effect of r-metHuGDNF on neuronal cell loss

**Figure 4:**
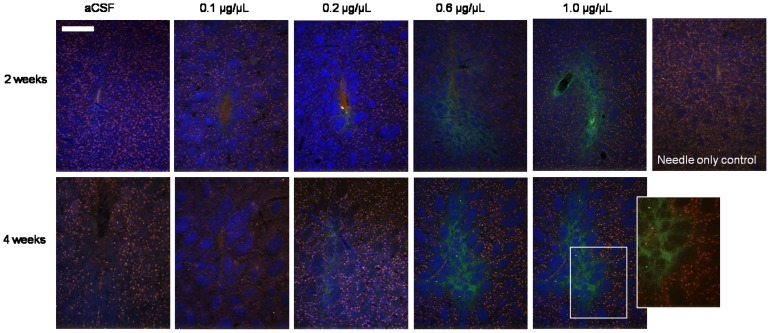
Immunostaining for NeuN to show neuronal cell loss. Neuonal cell loss was seen around the r-metHuGDNF infused area after 2 weeks and 4 weeks at 0.6 µg/µL and 1.0 µg/µL. A lack of NeuN (red) was generally visible where GDNF (green) had been infused at these concentrations (see inlay). Nuclei are marked in blue with the use of DAPI. Scale bar = 250 µm.

A lack of immunostaining for NeuN indicating neuronal cell loss in the r-metHuGDNF-infused area was observed in hemispheres infused with the two highest infused concentrations (0.6 µg/µL and 1.0 µg/µL) of r-metHuGDNF (Figure 4). By contrast, in needle only controls and in hemispheres infused with the lower concentrations (0.1 and 0.2 µg/µL) of r-metHuGDNF, no neuronal cell loss was detected, suggesting that the higher concentrations of r-metHuGDNF caused neuronal toxicity.

**Figure 5:**
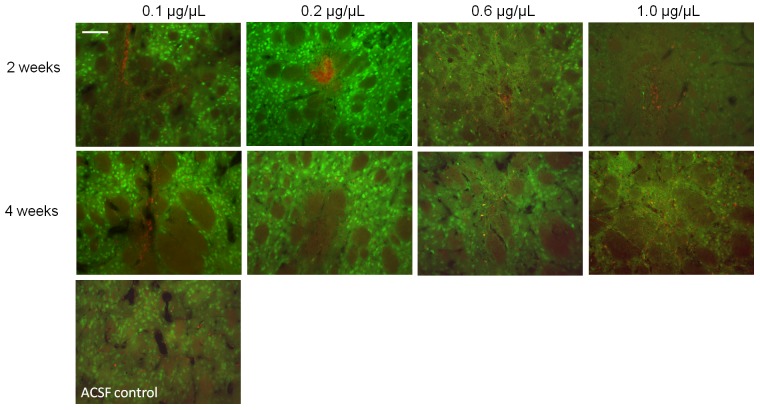
Immunostaining for activated caspase 3 to demonstrate apoptosis. An acute dose of GDNF (0.1, 0.2, 0.6 and 1.0 µg/µL) or aCSF vehicle control was infused into the striatum and tissue was collected after 2 weeks and 4 weeks. Representative images showing cells immuno-positive for activated caspase 3 (red) and NeuN (green). Scale bar = 100 µm.

In the hemispheres infused with the higher doses (0.6 and 1.0 µg/µL) of r-metHuGDNF, cells positive for activated caspase 3 co-localised with NeuN (Figure 5). No caspase 3/NeuN co-localisation was seen in aCSF infused hemispheres. Activated caspase 3 was observed within the needle track of brain hemispheres infused with lower doses of r-metHuGDNF (0.1 and 0.2 µg/µL) but fewer caspase 3 positive cells were seen in the surrounding tissue compared to the higher doses (0.6 and 1.0 µg/µL r-metHuGDNF).

#### 3.2.4 Alterations in synaptic and neurite outgrowth markers after administration of r-metHuGDNF

**Figure 6:**
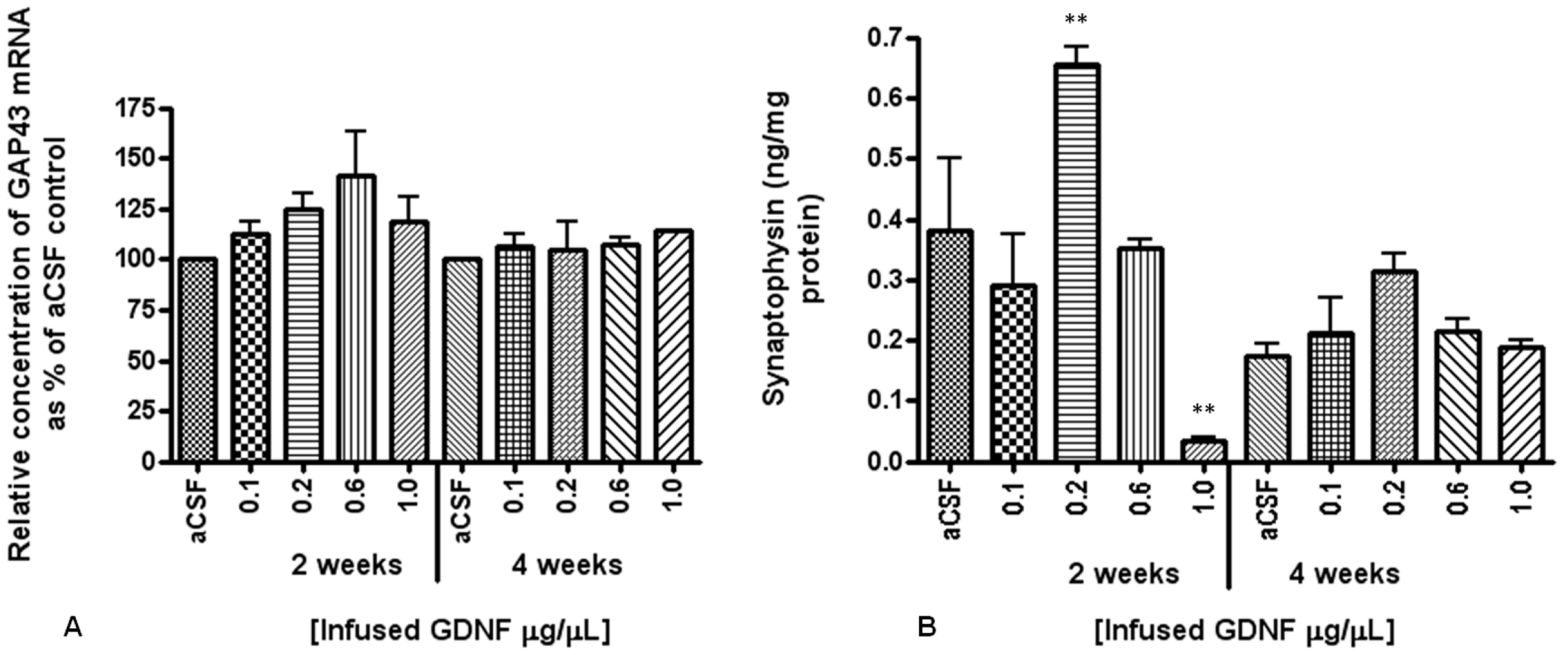
Expression of GAP43 mRNA and synaptophysin. Levels of GAP43 mRNA were analysed following Q-PCR analysis (A). Synaptophysin protein levels were measured using Sandwich ELISA (B). Each bar indicates the mean ± SD. **p<0.01, r-metHuGDNF infused hemispheres versus aCSF control infused hemispheres.

At both 2 and 4 weeks, there were no significant differences in GAP43 mRNA (a marker of neurite outgrowth), between aCSF infused control hemispheres and those infused with r-metHuGDNF. At 2 weeks, there was a non-statistically significant increase (41.5%) in GAP43 mRNA following infusion of 0.6 µg/µL r-metHuGDNF compared to aCSF (Figure 6A). Results from ELISA showed a substantial increase in synaptophysin protein following the infusion of 0.2 µg/µL r-metHuGDNF at both the 2 week (0.66±0.05 ng/mg of protein) and 4 week (0.31±0.05 ng/mg of protein) time-points compared to aCSF control (0.31±0.16 ng/mg of protein at 2 weeks, and 0.17±0.04 ng/mg of protein at 4 weeks), with the 2 week data reaching statistical significance (p<0.01, one-way ANOVA) (Figure 6B).

Synaptophysin ELISA also identified a significant reduction in this synaptic marker at 2 weeks following infusion of r-metHuGDNF at a concentration of 1.0 µg/µL compared to aCSF control (p<0.01 with one-way ANOVA). Taken in conjunction with the neuronal cell loss at 2 weeks post-infusion of the highest concentration of r-met HuGDNF, these findings are suggestive of synaptic toxicity resulting from the high infused r-metHuGDNF concentration.

## Discussion

Despite promising results in pre-clinical and phase I clinical trials, r-metHuGDNF is yet to fulfil its potential as a neurorestorative treatment for PD. The failure of r-metHuGDNF to provide clinical benefit in a phase II trial is likely to have been a consequence of inadequate distribution of r-metHuGDNF within the putamen [28] and accumulation of r-metHuGDNF at the site of infusion resulting in local toxicity [18]. CED has the potential to overcome the barriers to clinical benefit by achieving widespread, homogeneous and reflux-free drug distribution within the putamen [29]. It has also been suggested that an intermittent CED dosing regimen might maximise the volume of distribution within the putamen whilst minimising the risks of toxic accumulation and perivascular leakage of GDNF [30].

The aims of this study were to analyse the clearance and toxicity following acute infusion into rat striatum. The concentrations of 0.1, 0.2, 0.6, and 1.0 µg/µL were selected following previous clinical studies, which found significant improvement in functional outcomes following intraputamenal infusion of r-metHuGDNF at infused concentrations between 0.1 and 0.3 µg/µL [11].

The results show that infusion of r-metHuGDNF was not associated with an increase in microglial activation or gliosis compared with “needle only” controls at 2 and 4 weeks post-infusion. Both Iba1 and GFAP immunostaining was localised to the needle track, and the microglial response had diminished by 4 weeks post infusion.

In addition, we have found that after an acute delivery, r-metHuGDNF is substantially cleared within a 2 week period following CED, when infused at low concentrations (0.1 and 0.2 µg/µL). This finding suggests that local toxicity associated with accumulation of r-metHuGDNF could be prevented by employing an intermittent dosing regime with the dosing interval determined by both clearance and clinical effects. In addition, these low infused concentrations were not associated with neuronal loss, activated caspase 3 positive neurons or synaptic toxicity. At an infused concentration of 0.2 µg/µL r-metHuGDNF, the corresponding significant increase in synaptophysin and non-significant increase in GAP43 suggest that r-metHuGDNF could have neurorestorative effects even at this very low infused concentration. It should be noted that the antibodies employed for immunohistochemistry in this study may recognise short epitopes, and therefore degradation products of GDNF. Future studies could include HPLC analysis to examine the degradation of r-metHuGDNF to ensure that the protein remains active following infusion.

Significant levels of GDNF remained detectable within the striatum at 2 weeks following acute CED at the highest infused concentrations (0.6 and 1.0 µg/µL). Infusion of r-metHuGDNF at a concentration of 1.0 µg/µL was associated with a substantial decrease in synaptophysin, suggesting that high infused concentrations of r-metHuGDNF may cause synaptic toxicity. Infusions of r-metHuGDNF at the highest concentration were also associated with neuronal loss and activated caspase 3 suggesting that the persistence of r-metHuGDNF in the striatum resulted in neuronal toxicity. The long tissue half-life of r-metHuGDNF supports the future use of an intermittent infusion schedule, which may be preferable to continuous infusion by preventing r-metHuGDNF build up and point-source toxicity within the striatum. However, further pre-clinical studies may be required to determine whether an intermittent infusion regimen causes less neurotoxicity than the continuous infusions regimes previously employed in clinical trials. The translational relevance of repeated acute infusions of GDNF in a small animal model may be limited by the morbidity caused by repeated needle insertions into the striatum. Consequently, further pre-clinical studies would ideally be performed in a large animal model using an implantable CED catheter system [31].

Together, these findings suggest that high infused concentrations do not promote synaptogenesis, and that infusion of r-metHuGDNF at high concentrations (greater than 0.6 µg/µL) is associated with neuronal and synaptic toxicity. The results seen with acutely administered low infused concentrations of r-metHuGDNF imply that an intermittent infusion schedule of 0.1 or 0.2 µg/µL r-metHuGDNF could minimise the risk of toxic accumulation whilst achieving therapeutic benefit. An intermittent dosing schedule introduces the requirement for CED technology which facilitates repeated drug administrations for many years and perhaps for the lifetime of the patient. This requirement may be met by the recent development of a long term implantable CED catheter system which facilitates chronic intermittent drug delivery to the putamen [31].
